# Optimal surgical treatment for periprosthetic distal femoral fractures after total knee arthroplasty: a Bayesian-based network analysis

**DOI:** 10.1186/s13018-023-03586-y

**Published:** 2023-02-20

**Authors:** Peng Fu, Wenwei Liang, Zhenzhen Gao, Gang Chen, Weimin Fan

**Affiliations:** 1 Department of Orthopaedics, The First Affiliated Hospital of Nanjing Medical University, Nanjing, China; 2 Department of Clinical Oncology, The Second Affiliated Hospital of Jiaxing University, Jiaxing, China; 3 Department of Orthopaedics, The Second Affiliated Hospital of Jiaxing University, Jiaxing, China

**Keywords:** Periprosthetic fracture, Total knee arthroplasty, Locking compression plate, Retrograde intramedullary nailing, Distal femoral replacement

## Abstract

**Background:**

The surgical methods for periprosthetic distal femoral fractures (PDFFs) after total knee arthroplasty included locking compression plate (LCP), retrograde intramedullary nailing (RIMN), and distal femoral replacement (DFR). However, the optimal treatment remains controversial. We performed a network meta-analysis (NMA) to provide the optimal surgical method for PDFFs.

**Materials and methods:**

Electronic databases, including Embase, Web of Science, Cochrane Library, and PubMed, were searched for studies that compared LCP, RIMN, and DFR for PDFFs. The quality of the included studies was assessed according to the Newcastle–Ottawa scale. Pairwise meta-analysis was performed by Review Manager version 5.4. The NMA was conducted in Aggregate Data Drug Information System software version 1.16.5. We calculated odds ratios (ORs) and 95% confidence intervals (CIs) for postoperative complications and reoperations.

**Results:**

A total of 19 studies and 1198 patients were included, of whom 733 for LCP, 282 for RIMN, and 183 for DFR. Pairwise meta-analysis comparing LCP to RIMN and LCP to DFR showed no significant difference in complications and reoperations except that RIMN had a higher risk of malunion comparing to LCP (OR 3.05; 95% CI 1.46–6.34; *P* = 0.003). No statistically significant effects were found in the NMA of overall complications, infection, and reoperation. However, results of rank probabilities showed that DFR ranked best in overall complications and reoperation, RIMN ranked best in infection but worst in reoperation, and LCP ranked worst in infection and middle in reoperation.

**Discussion:**

We found similar complication rate and reoperation rate between LCP, RIMN, and DFR. The results of rank probabilities favored DFR, and further studies with high-level evidence are expected to verify the optimal surgical method for PDFFs.

**Level of evidence:**

Level II; network meta-analysis.

## Introduction

With the increasing rate of total knee arthroplasty (TKA) procedures [1], periprosthetic distal femoral fractures (PDFFs) have become more common, with an incidence of 3.5–5.5% [2–4]. As these fractures typically occur in older adults with poor bone quality and medical comorbidities, treatment is challenging and outcomes remain dismal, with a 1-year mortality rate up to 27% [5]. Surgical treatment is the mainstay treatment for PDFFs, including locking compression plate (LCP), retrograde intramedullary nailing (RIMN), and distal femoral replacement (DFR) [6].

Generally, the advantages of LCP include direct reduction and multi-angle fixation; however, RIMN requires less dissection of soft tissue, with its load-sharing structure being biomechanically superior to that of LCP [7]. In contrast to internal fixation, DFR allows early weight bearing and mobilization, which are critical for elderly patients [8–10]. Due to the complications associated with these surgical methods, the optimal treatment remains controversial [11]. Recent meta-analysis reported similar outcomes of LCP, RIMN, and DFR for PDFFs following TKA [12]. However, traditional meta-analysis can only compare two interventions simultaneously, while network meta-analysis (NMA) allows for the comparison of multiple interventions through combination of direct and indirect evidences.

Thus, we performed this NMA to compare surgical methods including LCP, RIMN, and DFR for PDFFs and figure out the optimal treatment with minimum complication and reoperation.

## Materials and methods

This meta-analysis was performed according to the PRISMA (Preferred Reporting Items for Systematic Reviews and Meta-Analyses) extension statement for NMA [13].

### Search strategy

Without limitations on study design, relevant articles were identified by an online literature search of databases, including Embase, Web of Science, Cochrane Library, and PubMed, from January 2000 to September 2021. Only English studies were eligible in the current study. The references of all eligible articles were screened for additional studies.

### Inclusion criteria

Inclusion criteria are as follows: (1) diagnosis of PDFFs after TKA; (2) comparison of at least two of the following surgical treatments: LCP, RIMN, and DFR; and (3) report of postoperative complications or reoperation rates. Two independent reviewers screened the identified studies after eliminating duplicates from the search results. If required, disagreements on the inclusion or exclusion of a study were resolved by discussion with a third reviewer acting as the adjudicator.

### Data extraction

Data extraction was performed by two independent reviewers, and the items included the first author’s name, year of publication, study design, age of patients, fracture classification, surgical methods, postoperative complications, and reoperation rate. The accuracy of all extracted data was confirmed before statistical analysis.

### Quality assessment

The quality of the included studies was strictly assessed by two independent reviewers according to the Newcastle–Ottawa scale [14].

### Statistics analysis

Pairwise meta-analysis was performed by Review Manager version 5.4 (Cochrane Collaboration). For dichotomous variables, we calculated odds ratios (ORs) and 95% confidence intervals (CIs). Heterogeneity among the included studies was estimated by chi-squared and *I*^2^ tests. The random effects model was employed except if *P* > 0.1 or *I*^2^ < 50%, which was considered less heterogeneous among these studies. In this case, the fixed effects model was applied. *P* < 0.05 was considered significant difference. We used Bayesian random effects regression model to perform the NMA in Aggregate Data Drug Information System (ADDIS) software version 1.16.5. *P* < 0.05 and 95% CIs exceeding null values were considered statistically different. Inconsistencies within the NMA were evaluated using inconsistency factors and variance calculations. We applied consistency and inconsistency model to evaluate the results. The consistency of direct and indirect evidence was evaluated based on node-splitting analysis, and *P* < 0.05 was considered a significant inconsistency. Convergence was assessed by the potential scale reduction factor (PSRF) value. PSPF values close to 1 are considered to be convergent. Furthermore, we assessed the ranking probability for each surgical method. A network plot was conducted by STATA version 14.0.

## Results

### Study characteristics

**Fig. 1:**
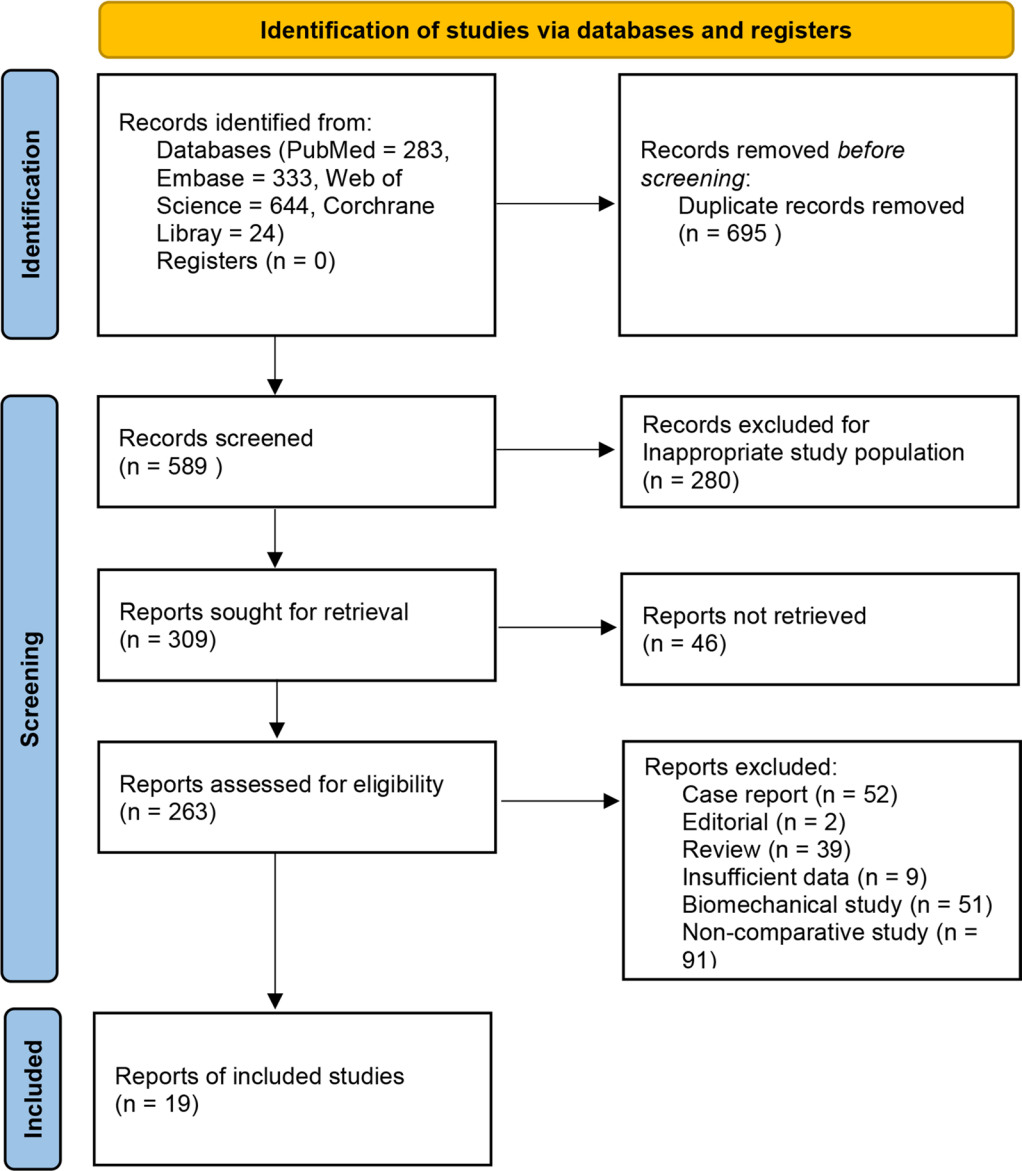
Flow diagram of study selection

**Fig. 2:**
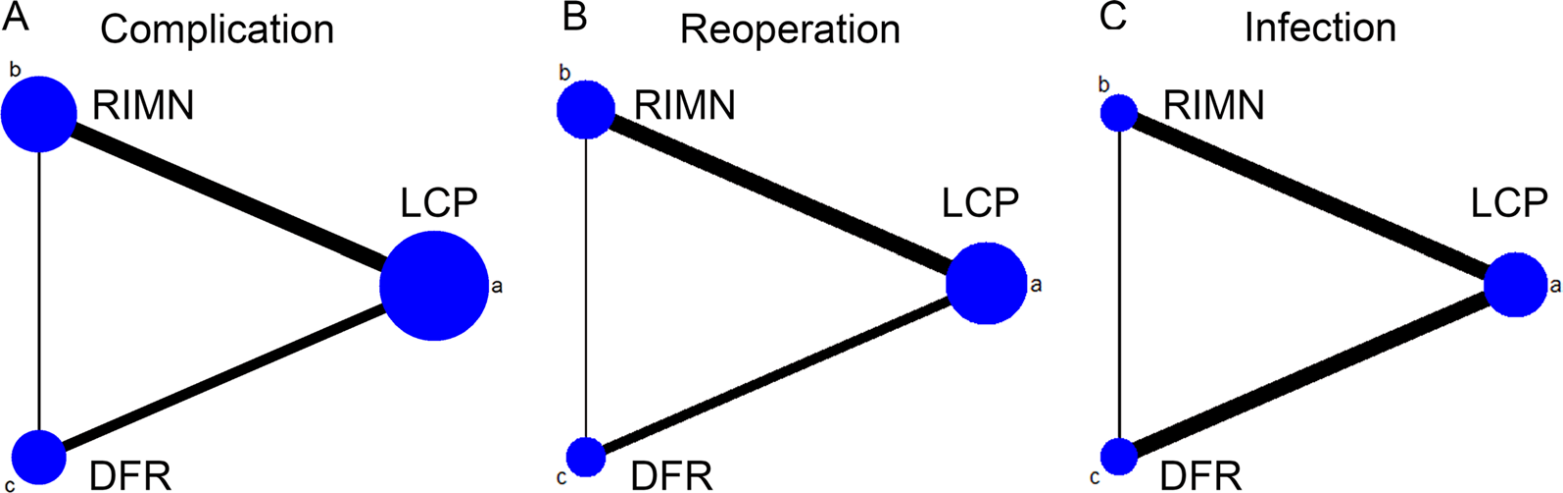
Network plot for overall complications (**A**), reoperation (**B**), infection (**C**)

Totally, 1284 studies were identified, of which 695 duplicated articles were excluded. After screening the remaining 589 articles, 19 studies were eligible for the meta-analysis [15–33]. A flow diagram of the search strategy and study selection is shown in Fig. 1. All eligible studies were retrospective case series; 11 studies compared LCP and RIMN, 6 studies compared LCP and DFR, and 2 studies compared LCP, RIMN, and DFR. Fracture classifications used in eligible studies included Rorabeck type [34] in 10 studies, Su type [35] in 5 studies, Orthopaedic Trauma Association classification [36] in 3 studies, Neer classification [37] in 1 study, and unified classification system (UCS) [38] in 1 study. A total of 1198 patients were included, of whom 733 for LCP, 282 for RIMN, and 183 for DFR. The characteristics of 19 eligible studies are listed in Table 1. The network plot is shown in Fig. 2.

**Table 1:** Characteristics of included studies

First author	Year	Intervention	Sample size	Mean age	Classification	Design
Large	2008	LCP:RIMN	29:7	74.8:74.1	Rorabeck	RS
Hou	2012	LCP:RIMN	34:18	75:77	OTA	RS
Aldrian	2013	LCP:RIMN	48:38	75.6	Su	RS
Horneff	2013	LCP:RIMN	11:46	69.5:68.3	Rorabeck	RS
Kilicoglu	2013	LCP:RIMN	9:7	76.7:68.6	Neer	RS
Gondalia	2014	LCP:RIMN	24:18	NA	OTA	RS
Meneghini	2014	LCP:RIMN	63:22	74:74	Rorabeck	RS
Leino	2015	LCP:DFR	39:29	78.8:79.2	Rorabeck	RS
Park	2016	LCP:RIMN	21:20	75:73.9	Rorabeck/Su	RS
Ruder	2017	LCP:DFR	35:23	78:83	NA	RS
Matlovich	2017	LCP:RIMN	36:19	75.7:75.4	Rorabeck	RS
Hoellwarth	2018	LCP:DFR	87:53	80:80.1	OTA/Su	RS
Gan	2018	LCP:DFR	8:7	67.9:76.7	Rorabeck	RS
Kyriakidis	2019	LCP:RIMN	31:29	76.1:82.1	Rorabeck	RS
Darrith	2020	LCP:RIMN:DFR	44:6:22	71.8:71.8:75.8	Rorabeck	RS
Guirao	2020	LCP:RIMN:DFR	48:40:9	75.1	Rorabeck	RS
Jennison	2020	LCP:DFR	42:13	80.5:79.2	UCS	RS
Gausden	2021	LCP:RIMN	74:23	76:75	Su	RS
Ross	2021	LCP:DFR	33:27	81.3:78.8	Su	RS

*NA* not available, *RS* retrospective study

### Methodological quality

The quality assessment scores of the included studies, as performed according to the Newcastle–Ottawa scale, are shown in Table 2.

**Table 2:** Newcastle–Ottawa scale of included studies

	Selection	Comparability	Exposure
Large	★★★	★	★★
Hou	★★★	★★	★★
Aldrian	★★★	★★	★★
Horneff	★★★	★★	★★
Kilicoglu	★★★	★	★★
Gondalia	★★★	★★	★★
Meneghini	★★★	★★	★★
Leino	★★★	★★	★★
Park	★★★	★★	★★
Ruder	★★★	★★	★★
Matlovich	★★★	★★	★★
Hoellwarth	★★★	★★	★★
Gan	★★★	★	★★
Kyriakidis	★★★	★★	★★
Darrith	★★★	★★	★★
Guirao	★★★	★	★★
Jennison	★★★	★★	★★
Gausden	★★★	★★	★★
Ross	★★★	★★	★★

### Pairwise meta-analysis

#### LCP versus RIMN

**Fig. 3:**
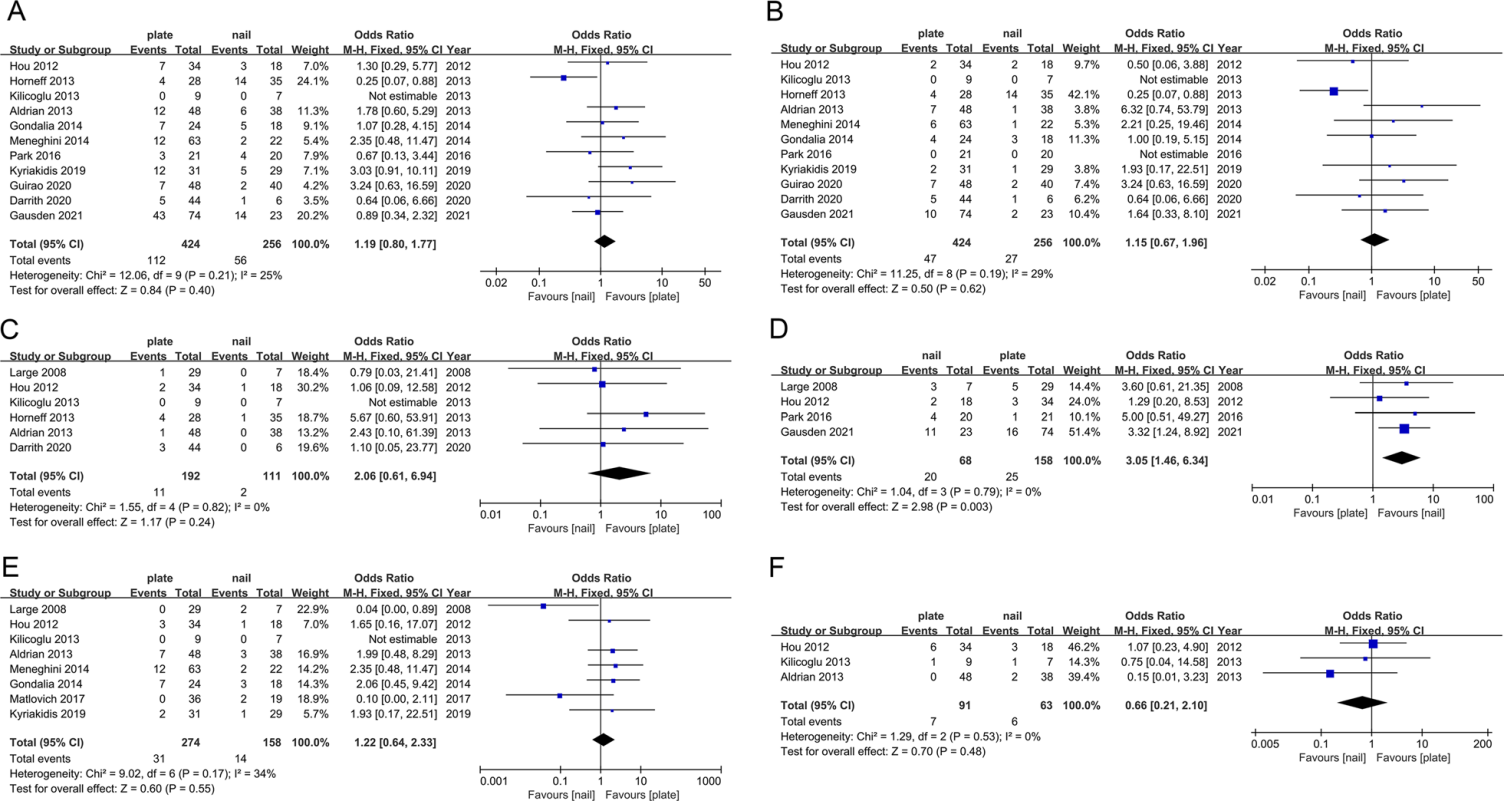
Forest plot of LCP vs. RIMN for overall complications (**A**), infection (**B**), nonunion (**C**), reoperation (**D**), malunion (**E**), 1-year mortality (**F**)

When comparing LCP and RIMN, the pooled results showed that the LCP group had a higher risk of overall complications (OR 1.19; 95% CI 0.80–1.77; *I*^2^ = 25%, *P* = 0.40, Fig. 3A), infection (OR 2.06; 95% CI 0.61–6.94; *I*^2^ = 0%, *P* = 0.24, Fig. 3B), nonunion (OR 1.22; 95% CI 0.64–2.33; *I*^2^ = 34%, *P* = 0.55, Fig. 3C), and reoperation (OR 1.15; 95% CI 0.67–1.96; *I*^2^ = 29%, *P* = 0.62, Fig. 3D), but no statistical significance was found. However, the risk of malunion was significantly higher in the RIMN group (OR 3.05; 95% CI 1.46–6.34; *I*^2^ = 0%, *P* = 0.003, Fig. 3E). No significant associations were found in the risk of 1-year mortality (OR 0.66; 95% CI 0.21–2.10; *I*^2^ = 0%, *P* = 0.48, Fig. 3F).

#### LCP versus DFR

**Fig. 4:**
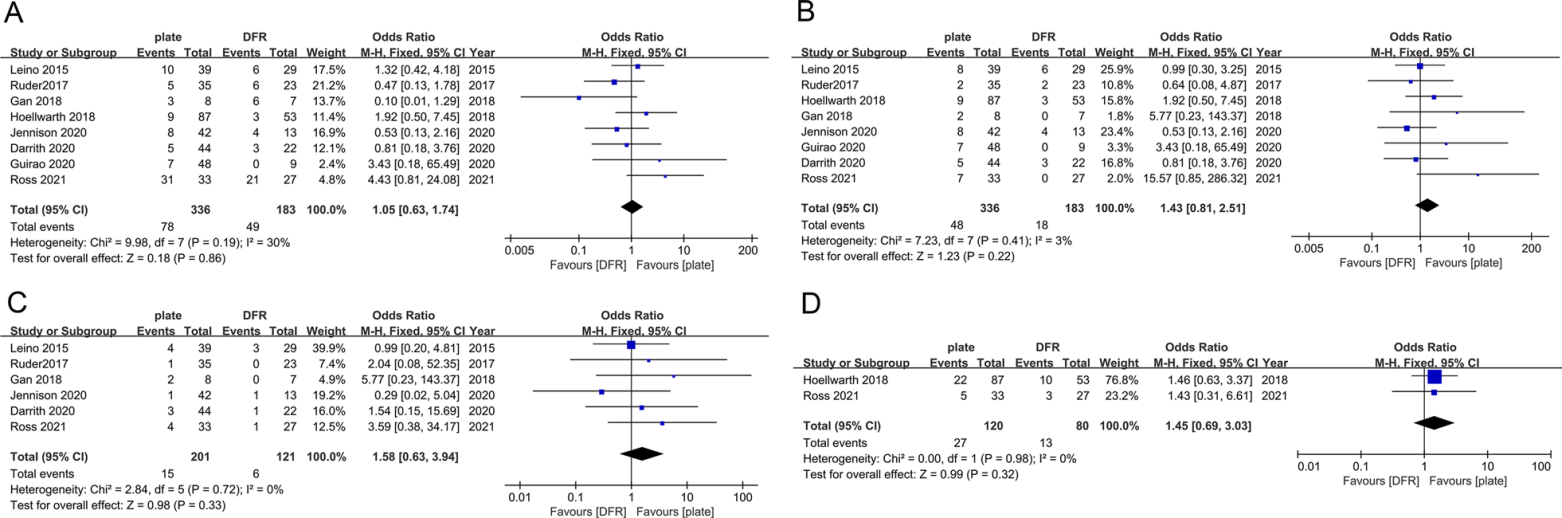
Forest plot of LCP vs. DFR for overall complications (**A**), infection (**B**), reoperation (**C**), 1-year mortality (**D**)

The pooled results showed that the LCP group had an increased risk of overall complications (OR 1.05; 95% CI 0.63–1.74; *I*^2^ = 30%, *P* = 0.86, Fig. 4A), infection (OR 1.58; 95% CI 0.63–3.94; *I*^2^ = 0%, *P* = 0.33; Fig. 4B), reoperation (OR 1.43; 95% CI 0.81–2.51; *I*^2^ = 3%, *P* = 0.22, Fig. 4C), and 1-year mortality (OR 1.45; 95% CI 0.69–3.03; *I*^2^ = 0%, *P* = 0.32, Fig. 4D), but no significant differences were found.

### Network meta-analysis

#### Inconsistency analysis

All the PSRF values were 1.00–1.02, revealing favorable convergence in the consistency and inconsistency model. As shown in Table 3, the median of the inconsistency factors was close to 0, and the median of the random effects standard deviation (SD) and inconsistency SD was roughly equal. Both showed favorable consistency of the evidence structure. Thus, we chose the consistency analysis for the NMA of overall complications, infection, and reoperation.

**Table 3:** Results of inconsistency factors and variance calculations for inconsistency analysis

Outcome	Inconsistency factors		Variance calculation	
	Cycle	Median (95%CI)	Random Effects Standard Deviation Median (95% CI)	Inconsistency Standard Deviation Median (95% CI)
Overall complications	DFR, LCP, RIMN	0.22 (− 1.18, 2.19)	0.98 (0.41, 1.91)	1.29 (0.05, 3.47)
Infection	DFR, LCP, RIMN	0.09 (− 1.65, 2.42)	0.64 (0.01, 1.64)	0.87 (0.05, 1.71)
Reoperation	DFR, LCP, RIMN	0.25 (− 1.19, 2.34)	1.24 (0.54, 2.31)	1.12 (0.04, 2.65)

#### Consistency analysis and rank probabilities

**Fig. 5:**
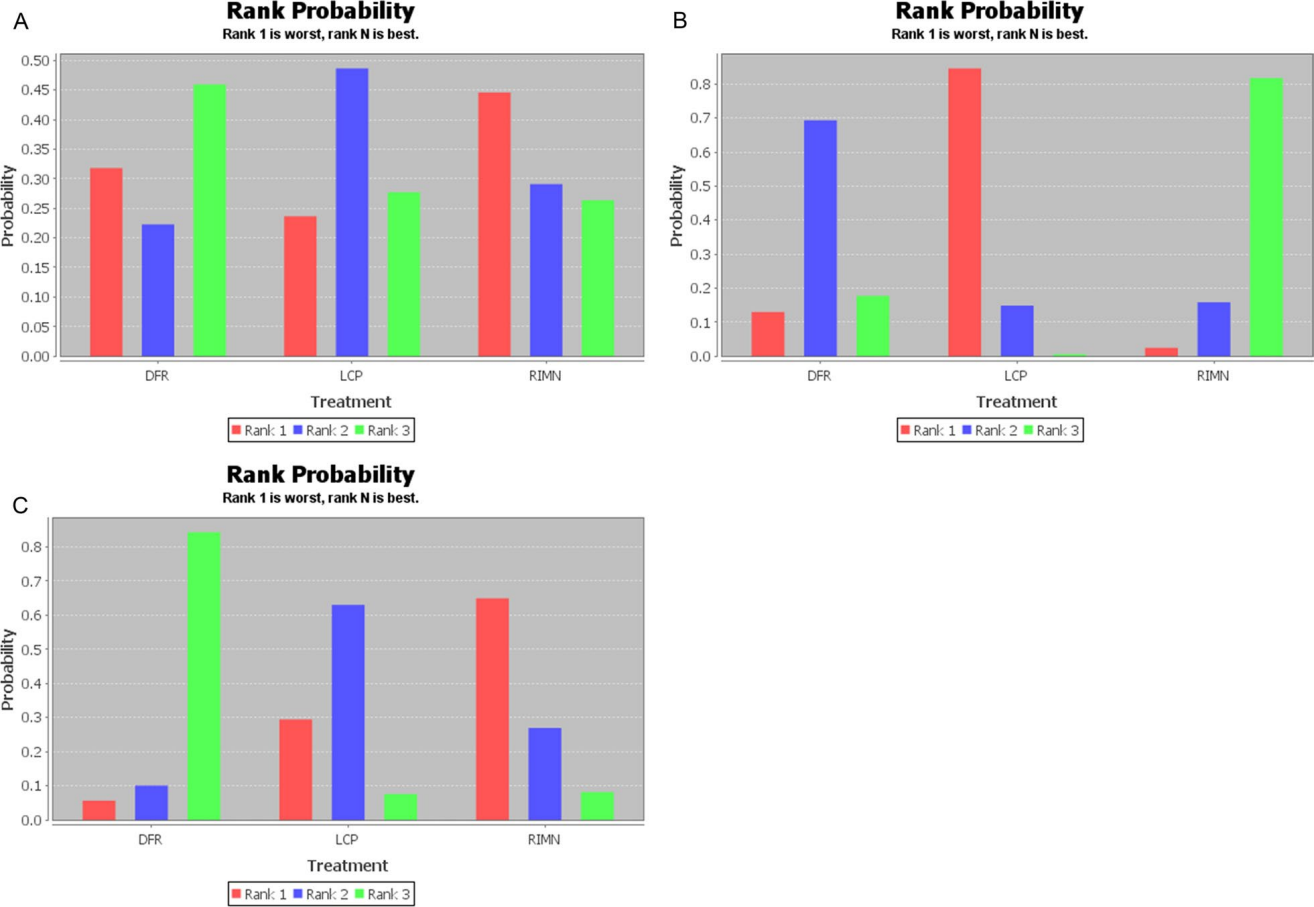
Results of rank probabilities for overall complications (**A**), reoperation (**B**), infection (**C**)

The number of studies used to perform the NMA of overall complications, infection, and reoperation were 18, 11, and 19, respectively. However, no statistically significant effects were found in the NMA as shown in Table 4. Results of rank probabilities (Table 5) indicated that DFR had highest score on rank 3 (46%) and RIMN had highest score on rank 1 (45%) in NMA for overall complications (Fig. 5A). Similarly, DFR had highest score on rank 3 (84%) and RIMN had highest score on rank 1 (65%) in NMA for reoperation (Fig. 5B). However, RIMN had highest score on rank 3 (82%) and LCP had highest score on rank 1 (85%) in NMA for infection (Fig. 5C).

**Table 4:** The network meta-analysis of consistency model

Overall complications	DFR		
	0.95 (0.36, 2.39)	LCP	
	0.87 (0.25, 2.87)	0.92 (0.41, 1.97)	RIMN
Infection	DFR		
	0.51 (0.12, 1.66)	LCP	
	2.71 (0.33, 30.81)	5.26 (0.96, 48.72)	RIMN
Reoperation	DFR		
	0.51 (0.14, 1.55)	LCP	
	0.41 (0.08, 1.69)	0.81 (0.31, 2.13)	RIMN

**Table 5:** Results of rank probabilities based on consistency model

Outcome	Invention	Rank1	Rank2	Rank3
Overall complications	DFR	0.32	0.22	0.46
	LCP	0.24	0.49	0.28
	RIMN	0.45	0.29	0.26
Infection	DFR	0.13	0.69	0.18
	LCP	0.85	0.15	0.01
	RIMN	0.02	0.16	0.82
Reoperation	DFR	0.06	0.10	0.84
	LCP	0.29	0.63	0.08
	RIMN	0.65	0.27	0.08

#### Node-splitting analysis

As shown in Table 6, the *P* values of node-splitting analysis in the NMA of overall complications, infection, and reoperation were 0.71, 0.14, and 0.52, respectively. The results indicated favorable consistency between direct and indirect evidence.

**Table 6:** Results of node-splitting analysis

Outcome	Name	Direct Effect	Indirect Effect	Overall	*P* value
Overall complications	DFR, RIMN	− 0.13 (− 2.54, 2.24)	0.28 (− 0.96, 1.57)	0.13 (− 1.02, 1.38)	0.71
Infection	DFR, RIMN	− 14.05 (− 51.74, 1.09)	− 0.89 (− 3.60, 1.54)	− 1.00 (− 3.43, 1.10)	0.14
Reoperation	DFR, RIMN	0.15 (− 2.50, 3.05)	1.08 (− 0.44, 2.86)	0.89 (− 0.53, 2.53)	0.52

## Discussion

Identifying optimal surgical treatment for PPDFs is challenging. In the current NMA study, through comparing surgical methods including LCP, RIMN, and DFR, we did detect similar overall complication rate and reoperation rate. However, results of rank probability showed the trend that DFR was related to minimum overall complication and reoperation, RIMN related to maximum complication and reoperation; meanwhile, LCP was in the middle.

Similar outcomes among these three surgical methods have been described by recent studies. Shin et al. reported similar outcomes of LCP and RIMN including operation time, Knee Society Score, time to union, nonunion rate, and revision rate [39]. The same results were detected by Magil et al. They included 10 retrospective studies comparing LCP and RIMN for analysis, and no significant differences were observed [40]. Another meta-analysis performed by Quinzi et al. [12] came into similar conclusion with major complication rate (19%, 16%, 19%) and reoperation rate (15%, 12%, 17%) among LCP, RIMN, and DFR, respectively, whereas the current study did find higher complication rate (27% for LCP, 24% for RIMN, 27% for DFR) since we recorded all documented complications for a comprehensive analysis. However, the reoperation and infection rate of DFR were lower in our study. We attributed this discrepancy to the included study conducted by Ross et al. [15], of which no reoperation was required and infection rate was limited to 3.7% (1/27) in DFR group.

In pairwise analysis, RIMN had a significantly increased risk of malunion comparing to LCP, confirming results reported by prior systematic reviews [41, 42]. They attributed the higher rate of malunion with RIMN to the difficulties obtaining the correct starting point and filling the wide metaphyseal flare. Besides, fewer distal locking screws with RIMN make it hard to maintain reduction. Pelfort et al. examined 23% hyperextension after RIMN procedure without radiological signs of femoral component loosening with patients presenting good function at 6-year follow-up [43]. Lee et al. reported 16% patients with RIMN developed malalignment with 100% union and mean Knee Society Score 81.5 [44]. Considering that malalignment was associated with higher failure rate of TKA [45], we cannot completely rule out the effect of malunion on function in the setting of PDFFs. Further studies focusing on relationship between severity of sagittal and coronal malunion and knee function are expected.

The major advantage of DFR is allowing early weight bearing. A systematic review reported the average time to full weight bearing of RIMN and LCP was 7.6 weeks and 15.8 weeks, respectively [46]. Rubinger et al. showed time to full weight bearing at 1.9 days for DFR [5]. However, 18–50% complication rate and 12.5–25% revision rate of DFR were reported in prior studies [47–49]. However, the current NMA found that DFR ranked best in overall complication and reoperation. Studies published within two years supported our results. Stancil et al. revealed 8% complications requiring revision of DFR and 88% patients were allowed immediate weight bearing with good function at 2-year follow-up [50]. Warschawski et al. found 10% complications with mean Knee Society Score 86.25 at average 4-year follow-up [51]. Chalmers et al. reported a 97% 5-year survivorship free from revision of contemporary DFR, and they also found that secondary DFR for PDFFs of failed internal fixation has a 5 times risk of revision relative to primary DFR [52]. We consider that the better outcome reported recently is related to improved surgical techniques. Considering the disadvantages of DFR including invasiveness, more technically challenging than internal fixation, subsequent periprosthetic fractures, extensor mechanism problems, and costs [53], we need more robust evidences to conclude that DFR is the optimal surgical method for PDFFs.

The major limitation of this NMA is that all included studies were retrospectively conducted with low-level evidence. Furthermore, various fracture classifications and implant designs used in the studies may have led to heterogeneity. Finally, clinical outcomes including operative time, knee function, and mortality were not included in the NMA due to insufficient data. Further prospective studies or randomized controlled trials are required.

## Conclusions

In summary, we found similar complication rate and reoperation rate between LCP, RIMN, and DFR. The results of rank probabilities favored DFR, and further studies with high-level evidence are expected to verify the optimal surgical method for PDFFs.

## Abbreviations

ADDIS: Aggregate Data Drug Information System
CIs: Confidence intervals
DFR: Distal femoral replacement
LCP: Locking compression plate
NMA: Network meta-analysis
ORs: Odds ratios
PDFFs: Periprosthetic distal femoral fractures
PSRF: Potential scale reduction factor
RIMN: Retrograde intramedullary nailing
SD: Standard deviation
TKA: Total knee arthroplasty
